# The Tumor Microenvironment-Dependent Transcription Factors AHR and HIF-1α Are Dispensable for Leukemogenesis in the Eµ-TCL1 Mouse Model of Chronic Lymphocytic Leukemia

**DOI:** 10.3390/cancers13184518

**Published:** 2021-09-08

**Authors:** Susanne Gonder, Anne Largeot, Ernesto Gargiulo, Sandrine Pierson, Iria Fernandez Botana, Giulia Pagano, Jerome Paggetti, Etienne Moussay

**Affiliations:** 1 Tumor Stroma Interactions, Department of Oncology, Luxembourg Institute of Health, L-1526 Luxembourg, Luxembourg; susanne.gonder@lih.lu (S.G.); anne.largeot@lih.lu (A.L.); ernesto.gargiulo@lih.lu (E.G.); sandrine.pierson@lih.lu (S.P.); iria.fernandezbotana@lih.lu (I.F.B.); giulia.pagano@lih.lu (G.P.); 2 Faculty of Science, Technology and Medicine, University of Luxembourg, L-4365 Esch-sur-Alzette, Luxembourg

**Keywords:** chronic lymphocytic leukemia, tumor microenvironment, AHR, HIF1α

## Abstract

**Simple Summary:**

Chronic lymphocytic leukemia (CLL) is the most common leukemia in Western countries, mostly affecting the elderly. The survival of leukemic cells depends on multiple soluble factors and on the stimulation of the BCR signaling pathway. Microenvironment-dependent transcription factors also contribute to CLL biology. Here, we generated new transgenic murine conditional knock-out models of CLL to study the role of the two transcription factors HIF-1α and AHR. Unexpectedly, we observed that both factors are dispensable for leukemia development in these models.

**Abstract:**

Chronic lymphocytic leukemia (CLL) is the most frequent leukemia in the elderly and is characterized by the accumulation of mature B lymphocytes in peripheral blood and primary lymphoid organs. In order to proliferate, leukemic cells are highly dependent on complex interactions with their microenvironment in proliferative niches. Not only soluble factors and BCR stimulation are important for their survival and proliferation, but also the activation of transcription factors through different signaling pathways. The aryl hydrocarbon receptor (AHR) and hypoxia-inducible factor (HIF)-1α are two transcription factors crucial for cancer development, whose activities are dependent on tumor microenvironment conditions, such as the presence of metabolites from the tryptophan pathway and hypoxia, respectively. In this study, we addressed the potential role of AHR and HIF-1α in chronic lymphocytic leukemia (CLL) development in vivo. To this end, we crossed the CLL mouse model Eµ-TCL1 with the corresponding transcription factor-conditional knock-out mice to delete one or both transcription factors in CD19+ B cells only. Despite AHR and HIF-1α being activated in CLL cells, deletion of either or both of them had no impact on CLL progression or survival in vivo, suggesting that these transcription factors are not crucial for leukemogenesis in CLL.

## 1. Introduction

In western countries, chronic lymphocytic leukemia (CLL) is the most common B cell malignancy in adults [1]. The disease occurs mostly in elderly patients and is characterized by the accumulation of mature monoclonal CD5+ B cells in peripheral blood, bone marrow, and lymphoid organs [2].

Genome instability is a well-known hallmark of cancer [3]. Indeed, alterations in the DNA and, by consequence, transcriptional programs increase the probability of neoplastic transformation and potentially enhance tumor immune evasion [4]. Also in CLL, gene mutations and dysfunction affecting transcriptional programs are essential prognostic factors for the disease outcome. In particular, given multiple its molecular abnormalities, CLL is considered a highly heterogeneous disease, including patients not requiring any therapy and patients having an aggressive course with poor response to therapy [5]. Major mutations in CLL affect many cellular components including inflammatory receptors (e.g., MYD88), kinases such as MAPK (e.g., BRAF), NF-kB-related molecules (e.g., BIRC3), transcription (e.g., EGR2 and NOTCH1) and splicing factors (e.g., SF3B1), DNA damage and cell cycle control factors (e.g., ATM and TP53) [6]. These genetic abnormalities have supported the generation of novel agents against CLL which have been translated into the clinical practice towards a more targeted treatment strategy for patients [7]. Treatments for CLL patients include chemotherapy, chemoimmunotherapy, and small molecules mostly targeting important signaling pathways in CLL cells (e.g., BCR and BCL2) [8]. Even after a long remission phase, patients can relapse and develop resistance to treatments. For this reason, investigating new strategies for the development of treatments against CLL is key for the improvement of patients’ health.

In the past decades, the transcription factors (TFs) aryl hydrocarbon receptor (AHR) and hypoxia inducible factor-1α (HIF-1α) have been recognized to strongly impact cancer progression and escape mechanisms [9,10,11,12]. AHR is a ligand-activated TF involved in many biological processes, such as cell division, quiescence, and inflammation [13]. The most known agonist of AHR is 2,3,7,8 tetrachlorodibenzo-*p*-dioxin (TCDD); however, more importantly for the field of tumor immunology, is the endogenous agonist kynurenine, which originates from tryptophan, in a reaction catalyzed by indoleamine 2,3-dioxygenase 1 (IDO1) and tryptophan 2,3-dioxygenase 2 (TDO2) [14]. Importantly, AHR activation by this pathway has been shown to contribute to immune escape mechanisms leading to an immunosuppressive tumor microenvironment (TME) [15,16].

On the other hand, HIF-1α is an oxygen-sensitive TF, stabilized in a hypoxic environment and by certain non-canonical mechanisms, called pseudohypoxia [17]. Here, for example, metabolites of the Krebs cycle can block prolyl hydroxylases (PHDs), which under normoxic conditions, hydoxylate HIF-1α in order to trigger ubiquitination by the von Hippel–Lindau protein (pVHL) and to start proteasomal degradation [18]. HIF-1α is known to be involved in tumor progression and is mostly associated with poor patient’s outcome [19]. Interestingly, AHR and HIF-1α share the dimerization partner ARNT/HIF1β, showing linked processes of tumor progression, metabolic pathways, and vascular development [20]. Moreover, these TFs have an impact on proliferation and functions of B lymphocytes and CLL cells [21,22]. Villa et al. showed that AHR is necessary for B cell proliferation and that cyclin O is directly impacted by AHR deficiency [23]. Furthermore, AHR seems to contribute to the transcriptional program of IL-10-producing regulatory B cells [24], which is a B cell subset sharing regulatory functions with CLL cells [25]. On the other hand, HIF-1α has been already described as highly involved in CLL pathogenesis and in the interaction with the TME. In 2016, Valsecchi and colleagues showed that this interaction is regulated by HIF-1α and promotes tumor survival and tumor propagation [26]. Indeed, HIF-1α is overexpressed in leukemic cells from TP53-disrupted patients and, thus, would be an interesting target for new therapies for CLL [27].

Currently, the impact of AHR and HIF1α in CLL development in vivo has not been fully evaluated. In this article, we generated conditional knock-out mice for Ahr, Hif1a, or both genes restricted to B cells of the Eµ-TCL1 transgenic mouse model, the most extensively used and studied animal model for CLL [28].

## 2. Materials and Methods

### 2.1. Mice

Animals were kept in a specific pathogen-free facility, and animal experiments were approved by the internal Animal Welfare Structure and the Luxembourg Ministry for Agriculture. Mice were treated in accordance with the European Union guidelines.

C57BL/6 mice were purchased from Janvier Labs (France). To generate B cell-specific *Ahr*-, *Hif1a*-, and *Ahr-Hif1a*-deficient mice, *Ahr^fl/fl^*(#006203), *Hif1a^fl/fl^* (#007561), and *Ahr^fl/fl^ Hif1a^fl/fl^* were crossed with *CD19^WT/Cre^* (#006785) mice. These mice were bred with Eµ-TCL1 mice to introduce the TCL1 oncogene under the IgVH promoter. These mice were described previously [28]. The mice were bred and maintained on a C57BL/6 background, and Eµ-TCL1 *CD19^Cre/WT^Hif1a^WT/WT^Ahr^WT/WT^* mice were used as WT controls. CLL progression in the transgenic mouse models was monitored over several months by determining the percentage of CD5+CD19+ CLL cells in peripheral blood mononuclear cells (PBMC) by flow cytometry analysis on a Cytoflex (Beckman Coulter, Brea, CA, USA) using CD19-APC and CD5-PE (Biolegend, San Diego, CA, USA). Mice reaching the humane endpoint were euthanized by cervical dislocation. All deaths unrelated to leukemia were excluded from this study. To perform adoptive transfer in healthy conditional knock-out mice, CLL cells were isolated from the spleen of diseased Eµ-TCL1 mice. Then, 10 × 10^6^ CLL cells were injected intravenously in 100 µL of DMEM, and CLL progression was followed by weekly bleeding, as described previously.

### 2.2. B Cell Isolation

B cells were purified from the spleen by negative selection using the MojoSort™ Mouse Pan B Cell Isolation Kit II (Biolegend, San Diego, CA, USA) following the manufacturer’s instructions. The isolated B cell population contained at least 90% of CD19+CD5+-double positive cells. B cells were cultured in RPMI supplemented with 10% FBS, 50 µM 2-β-mercaptoethanol, 100 U/mL penicillin, and 100 µg/mL streptomycin. To mimic hypoxia, 150 µM cobalt chloride (Sigma-Aldrich, Burlington, MA, USA) was added, and the cells were cultured overnight at 37 °C and 5% CO_2_. In order to activate AHR, the cells were incubated with 250 nM FICZ (Sigma-Aldrich, Burlington, MA, USA) for 2 hours at 37 °C and 5% CO_2._

### 2.3. Validation of the Models at the gDNA, RNA, and Protein Levels

Genomic DNA was extracted using NucleoSpin Tissue Mini kit for DNA from cells and tissue (Macherey-Nagel, Düren, Germany) following the manufacturer’s instructions. To perform the PCR on gDNA for *Ahr*, the PCR mix 2x M-PCR OPTI™ Mix (Bimake, Houston, TX, USA) was used as described in the instructions, and the specific primer sequences were: 5′-GTCACTCAGCATTACACTTTCTA-3′, 5′-CAGTGGGAATAAGGCAAGAGTGA-3′, and 5′-GGTACAAGTGCACATGCCTGC-3′. The use of these three primers enables the detection of wild-type (106 bp), floxed (140 bp), and excised (180 bp) alleles. The amplification of *Ahr* was performed with the following program: 94 °C for 5 min, 30 cycles of 94 °C for 15 s, 56 °C for 15 s, and 72 °C for 30 s. After amplification, the product was run on a 3% agarose gel with SYBR™ Safe DNA Gel Stain (ThermoFisher, Waltham, MA, USA) and visualized by Image Quant Las 4000 (GE Healthcare, Chicago, IL, USA). For *Hif1a*, we used the HIF1a TaqMan copy number assay Mm00375032_cn with the TaqMan™ Copy Number Reference Assay, mouse, Tfrc (Thermo Scientific, Waltham, MA, USA). Quantitative PCRs were performed using Takyon Low Rox Probe 2X mastermix (Eurogentec, Seraing, Belgium) according to manufacturer’s instructions. The qPCRs were run on the QuantStudio™ 3 or 5 (Applied Biosystems, Waltham, MA, USA) with the following program for the Taqman assay: 50 °C 2 min, 95 °C for 10 min, 40 cycles of 95 °C for 15 s, and 60 °C for 1 min.

RNA from B cells was extracted using Nucleozol reagent and the NucleoSpin^®^ RNA Set for NucleoZOL (Macherey-Nagel, Düren, Germany) and quantified using the Nanophotometer N60 (Implen, München, Germany). The complementary DNA was synthesized from 500 ng of RNA by using FastGene Scriptase II cDNA 5x ReadyMix (Nippon Genetics, Düren, Germany) according to the manufacturer’s instructions. Quantitative PCRs were performed using Takyon for SYBR Assay Low Rox or Takyon Low Rox Probe 2X mastermix (Eurogentec, Seraing, Belgium). The specific primer sequences were: *Ahr*: 5′-AGCCGGTGCAGAAAACAGTAA-3′ and 5′-AGGCGGTCTAACTCTGTGTTC-3′; *Hprt*: 5′-TGGATACAGGCCAGACTTTGTTF-3′ and 5′-CAGATTCAACTTGCGCTCATC-3′; *Tbp*: 5′-GTCATTTTCTCCGCAGTGCC-3′ and 5′-GCTGTTGTTCTGGTCCATGAT-3′. For *Hif1a*, we used the HIF1a TaqMan assay Mm00375032_cn (Thermo Scientific, Waltham, MA, USA). The qPCR was performed similarly as described above.

The comparative Ct method was used to calculate gene expression relative to housekeeping gene *Hprt* and *Tpb*.

For protein isolation, cultured B cells were washed twice with ice-cold PBS, and proteins were extracted using RIPA buffer including the cOmplete™ Protease Inhibitor Cocktail (Roche, Basel, Switzerland) and the Phosphatase Inhibitor Cocktail 2 and 3 (Sigma-Aldrich, Burlington, MA, USA). Then, 10 µg of cell lysates were resolved on 10% SDS-PAGE and transferred to a nitrocellulose membrane. To confirm equivalent loading between lanes, a Ponceau red staining was performed. Membranes were incubated in 1x PBS-T (0.1%) and fat-free dry milk (5%, Roth) blocking buffer during one hour at room temperature. Membranes were then incubated with primary antibodies against HIF-1α (#36169, Cell Signaling), AHR (#694502, Biolegend, San Diego, CA, USA), and beta-actin-HRP (#A3854, Sigma-Aldrich, Burlington, MA, USA) in blocking buffer at 4 °C overnight. Membranes were washed three times in 1xPBS-T (0.1%) for 10 min each time. Secondary antibodies coupled to HRP were from Jackson ImmunoResearch. For detection, the ECL western blot detection kit was purchased from Amersham, and the radiographic films from Santa Cruz Biotechnology.

### 2.4. Sample Preparation for RNA Sequencing

Control C57BL/6 and diseased Eµ-TCL1, or Eµ-TCL1 *CD19 ^Cre/WT^ Hif1α^fl/fl^*, Eµ-TCL1 *CD19^Cre/WT^ Ahr^fl/fl^*, Eµ-TCL1 *CD19^Cre/WT^ Hif1a^fl/fl^ Ahr^fl/fl^*, and the corresponding control mice Eµ-TCL1 *CD19^Cre/WT^ Hif1α^WT/WT^Ahr^WT/WT^* were euthanized at humane endpoint by CO_2_ inhalation or cervical dislocation. Spleens were collected, and single cell suspensions were prepared as previously described [29,30]. CD19+ B cells or CD5+CD19+ CLL cells were sorted with a BD FACSAria III at 4 °C. Then, 1–5 × 10^6^ sorted cells were centrifuged and resuspended in 500 µL of Nucleozol reagent. Total cellular RNA was extracted using the NucleoSpin RNA Set for NucleoZOL and eluted in 30 µL of RNAse-free water. Libraries were prepared with the QuantSeq 3’ mRNA-Seq Library Prep Kit FWD for Illumina (Lexogen, Vienna, Austria) with dual indexing, according to the manufacturer’s instructions, with the addition of UMI. Barcoded samples were diluted, pooled, loaded onto a NextSeq 500/500 Mid Output flowcell or a NovaSeq SP flowcell (Illumina, San Diego, CA, USA), and single-end sequencing was performed using a NextSeq 550 or a NovaSeq 6000 system (Illumina, San Diego, CA, USA).

### 2.5. RNA Sequencing Analysis

After initial QCs using FastQC (www.bioinformatics.babraham.ac.uk/projects/fastqc/, accessed on 19 March 2021) and FastQ Screen (www.bioinformatics.babraham.ac.uk/projects/fastq_screen/, accessed on 19 March 2021), fastq files were processed using a local Snakemake workflow including the following main steps. First, raw reads were trimmed from their UMI index, poly A, and adapter sequences using a combination of dedicated scripts and cutadapt (v2.10). Next, the filtered reads were submitted for mapping (STAR v2.5.3a) on the Mouse Reference genome (GRCm38). Collapsing of reads originating from the same fragment was achieved with umi_tools (v 1.0.0), and counting was performed with featureCounts (subread v2.0.0).

Counts were filtered and transformed with EdgeR for clustering and principal component analysis (PCA). For k-means clustering, the 2500 most variable genes were included, and 6 clusters were defined according to the elbow method (online tool iDEP.93). Top genes were mapped with the dimension reduction algorithm t-SNE. Differential expression of genes across C57BL/6 and Eµ-TCL1 samples (DEG) was assessed using the DESeq2 package, and FDR < 0.05 and log2 fold change cut-off of 1 were imposed. Raw and processed data were deposited in the NCBI GEO database (GSE175564). 

Differential expression of genes across Eµ-TCL1 *CD19^Cre/WT^ Hif1a^WT/WT^Ahr^WT/WT^*, Eµ-TCL1 *CD19^Cre/WT^Hif1α^fl/fl^*, Eµ-TCL1 *CD19^Cre/WT^Ahr^fl/fl^*, Eµ-TCL1 *CD19^Cre/WT^ Hif1a^fl/fl^ Ahr^fl/fl^* samples (DEG) was assessed using the EdgeR package, and FDR < 0.05 and log2 fold change cut-off of 1 were imposed. Pheatmap and EnhancedVolcano packages were used for data visualization. Raw and processed data were deposited in the NCBI GEO database (GSE179196). 

### 2.6. Murine B Cell-Specific Ahr-Induced Gene Signature

The murine B cell-specific *Ahr*-induced gene signature (mouse_B_Ahr) was defined based on the gene expression data from Kovalova et al. (2017, GSE80953). Briefly, CEL files corresponding to murine B cells treated with vehicle (VH) or TCDD (2,3,7,8-tetrachlorodibenzo-p-dioxin) for 4 h, 8 h, or 24 h were loaded into the Transcriptome Analysis Console (TAC 4.0, Applied Biosystems), and the 153 genes induced by TCDD (fold change >1.5 and FDR < 0.05) at any time points (TCDD vs. VH) were included in the signature.

### 2.7. Gene Set Enrichment Analysis

We performed GSEA using the GSEA Broad Institute stand-alone software (http://www.gsea-msigdb.org/gsea/index.jsp, accessed on 20 April 2021). Gene counts from both groups (C57BL/6 and Eµ-TCL1) were loaded into GSEA according to recommendations. Data were tested against the above-described mouse_B_Ahr signature and the GSEA Hallmarks signatures. Normalized enrichment scores (NES) and FDR < 0.1 were taken into consideration.

### 2.8. Transcription Factor Enrichment Analysis

DEGs significantly upregulated in leukemic B cells from Eµ-TCL1 were subjected to transcription factor enrichment analysis with the online tool ChEA3 (Ma’ayan Laboratory, Icahn School of Medicine at Mount Sinai) predicting transcription factors associated with user-input sets of genes. DEGs were also submitted for Integrated System for Motif Activity Response Analysis performed with the free online ISMARA tool that recognizes the most important transcription factors impacting on the transcription profiles of a set of samples. The network of protein–protein interactions for transcription factors was constructed with string-db (v11.0, STRING Database).

### 2.9. Statistical Analysis

**Figure 1:**
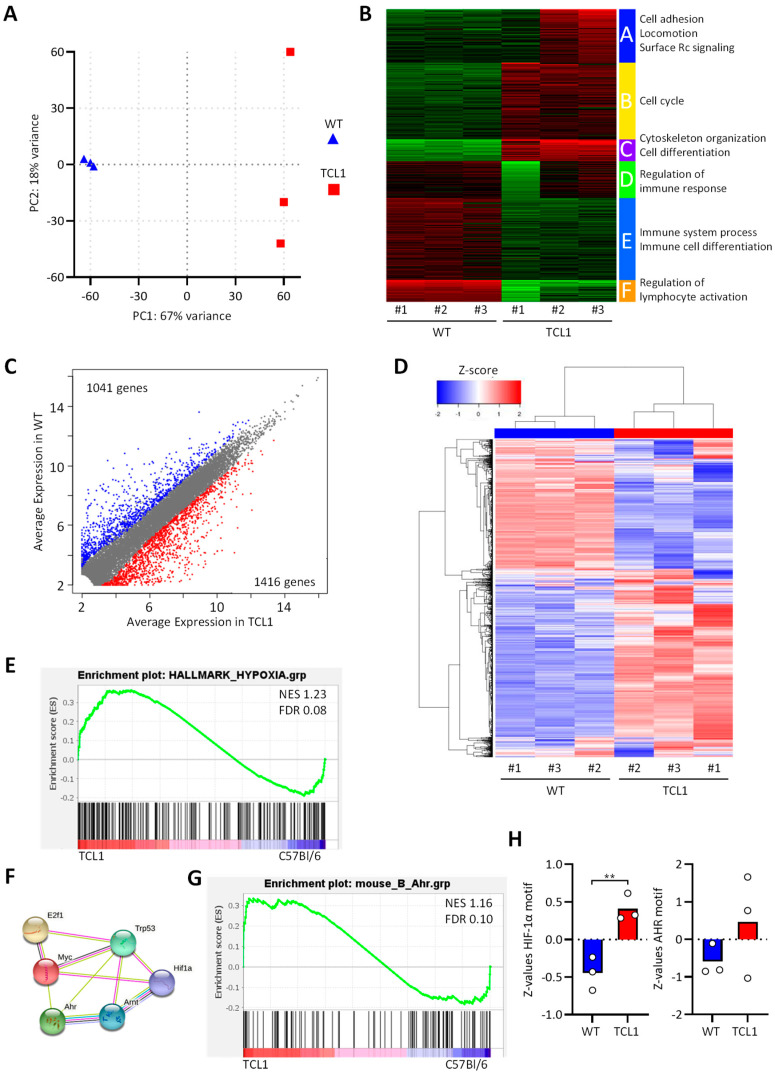
RNA sequencing of B cells from C57BL/6 and Eµ-TCL1 mice. Splenic B cells were FACS-sorted from three mice of each genotype, and mRNA was sequenced. (**A**) Principal component analysis of individual animals. (**B**) K-means clustering and Gene Ontology enrichment analysis. (**C**) Scatterplot depicting the expression of genes in the groups. (**D**) Unsupervised hierarchical clustering showing 1416 genes upregulated and 1041 genes downregulated in TCL1. (**E**,**G**) Gene Set Enrichment Analysis showing the enrichment of hypoxia (**E**) and AHR (**G**) signatures in Eµ-TCL1 versus C57BL/6 mice. (**F**) Protein–protein interactions network (STRING) for transcription factors involved in enriched hallmark pathways (GSEA). (**H**) Transcription factor activity (Z-values, ISMARA) for HIF-1α and AHR motifs in WT and TCL1 B cells.

**Figure 2:**
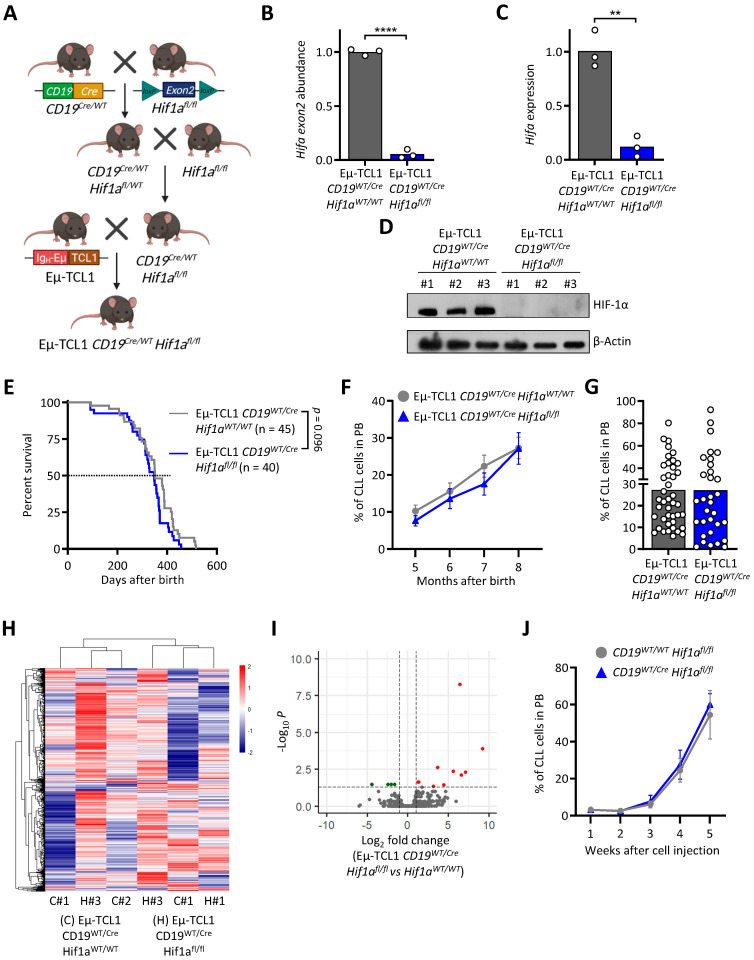
Knocking out HIF-1α does not show an effect on the leukemogenesis of CLL cells in the murine Eµ-TCL1 model. (**A**) Scheme of the breeding strategy to generate Eµ-TCL1 *CD19^Cre/WT^ Hif1a^fl/fl^* (cKO). (**B**–**D**) Validation of the knock-out of exon 2 of *Hif1a* in isolated B cells from cKO mice incubated with CoCl_2_ at the DNA (**B**), RNA (**C**), and protein (**D**) levels compared to control mice (n = 3). ** *p* < 0.01, **** *p* < 0.0001. (**E**) Survival curve of cKO compared to control mice shows no significant difference. (**F**–**G**) Flow cytometry analysis of CLL cells (CD19+ CD5+) in the peripheral blood (PB) of cKO and control mice over time (Ctrl: n = 45, cKO: n = 39) (**F**) and at month 8 (Ctrl: n = 42, cKO: n = 35) (**G**). (**H**–**I**) CLL cells from cKO and control mice (n = 3) were subjected to RNA sequencing. (**H**) Unsupervised hierarchical clustering showing the top 10,000 most expressed genes. (**I**) Volcano plot showing DEG between cKO and control mice. (**J**) CLL development of the adoptive transfer of Eµ-TCL1 CLL cells in CD19*^Cre/WT^ Hif1a^fl/fl^* mice (Ctrl: n = 5, cKO: n = 6).

**Figure 3:**
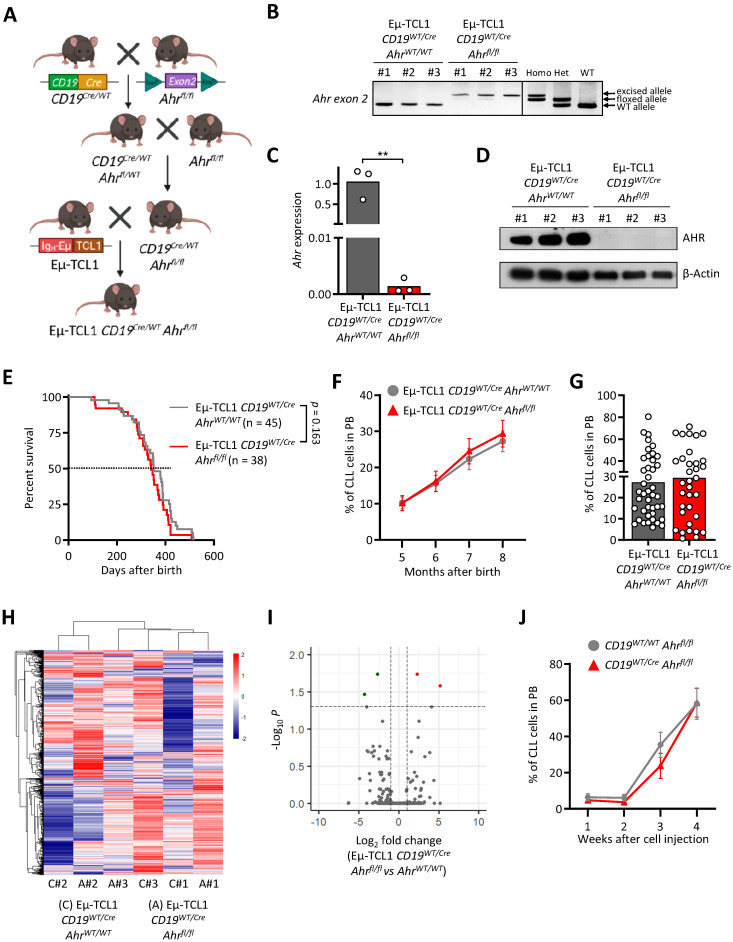
AHR knock-out does not influence CLL development in the murine Eµ-TCL1 model. (**A**) Scheme of the breeding strategy to generate Eµ-TCL1 CD19*^Cre/WT^ Ahr^fl/fl^* (cKO) mice. (**B**–**D**) Validation of the knock-out of exon 2 of *Ahr* in isolated B cells from cKO mice incubated with FICZ at the DNA (**B**), RNA (**C**), and protein (**D**) levels compared to control mice (n = 3). ** *p* < 0.01. (**E**) Survival curve of cKO compared to control mice shows no significant difference. (Ctrl: n = 45, cKO: n = 38). (**F**,**G**) Flow cytometry analysis of CLL cells (CD19+ CD5+) in the peripheral blood (PB) of cKO and control mice over time (Ctrl: n = 45, cKO: n = 38) (**F**) and at month 8 (Ctrl: n = 42, cKO: n = 34) (**G**). (**H**,**I**) CLL cells from cKO and control mice (n = 3) were subjected to RNA sequencing. (**H**) Unsupervised hierarchical clustering showing the top 10,000 most expressed genes. (**I**) Volcano plot showing DEG between cKO and control mice. (**J**) CLL development of the adoptive transfer of Eµ-TCL1 CLL cells in *CD19^Cre/WT^ Ahr^fl/fl^* mice (Ctrl: n = 5, cKO: n = 5).

**Figure 4:**
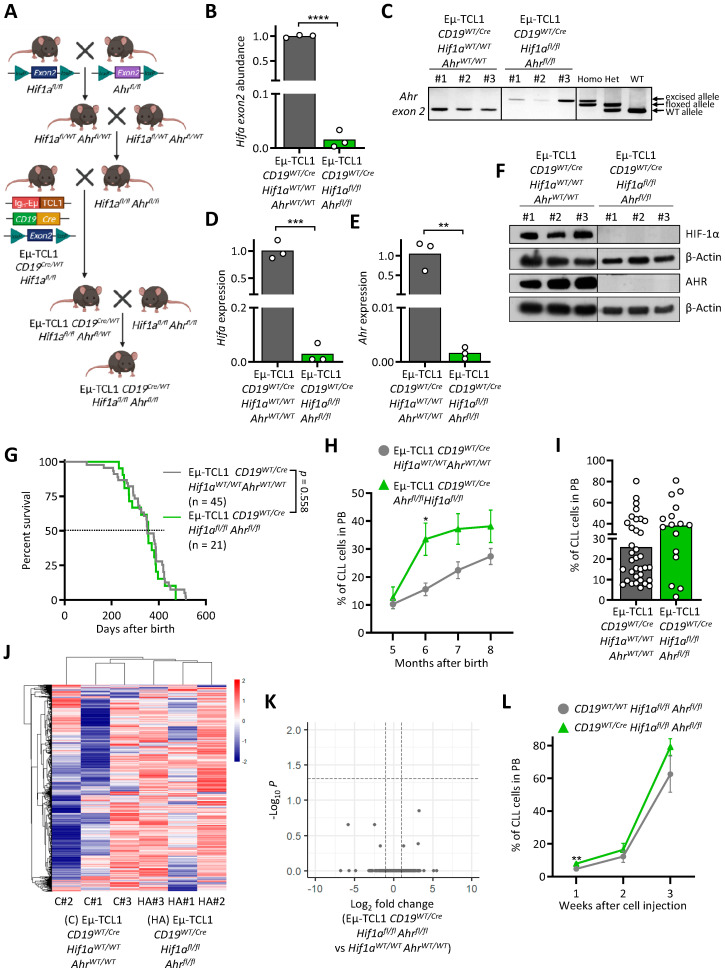
Double knock-out of AHR and HIF-1α does not appear to have an impact on the development of neoplastic B cells. (**A**) Scheme of the breeding strategy to generate Eµ-TCL1 *CD19^Cre/WT^ Hif1a^fl/fl^Ahr^fl/fl^* (cDKO) mice. (**B**–**F**) Validation of the knock-out of exon 2 of *Hif1a* and *Ahr*, respectively, in isolated B cells from cDKO mice incubated with CoCl_2_ and FICZ at the DNA (**B**,**C**), RNA (**D**,**E**), and protein (**F**) levels compared to control mice (n = 3). ** *p* < 0.01, *** *p* < 0.001, **** *p* < 0.0001. (**G**) Survival curve of cDKO compared to control mice shows no significant difference. (Ctrl: n = 45, cDKO: n = 21). (**H**,**I**) Flow cytometry analysis of CLL cells (CD19+ CD5+) in the peripheral blood (PB) of cDKO and control mice over time (Ctrl: n = 45, cDKO: n = 21) (**H**) and at month 8 (Ctrl: n = 35, cDKO: n = 16) (**I**). (**J**,**K**) CLL cells from cDKO and control mice (n = 3) were subjected to RNA sequencing. (**L**) Unsupervised hierarchical clustering showing the top 10,000 most expressed genes. (**I**) Volcano plot showing DEG between cDKO and control mice. (**J**) CLL development of the adoptive transfer of Eµ-TCL1 CLL cells in CD19*^Cre/WT^ Hif1a^fl/fl^ Ahr^fl/fl^* mice (Ctrl: n = 4, cDKO: n = 4).

Statistical analysis was performed using GraphPad Prism software (version 9.1.2). The unpaired t test was used for Z-values analyses and qPCRs (Figure 1H, Figure 2B, Figure 2C, Figure 3C and Figure 4B,D,E). The log-rank test was used for the survival curves (Figure 2E, Figure 3E and Figure 4G). For the percentage of CLL cells in the transgenic mouse models overtime (Figure 2F, Figure 3F, and Figure 4H), we used mixed-effects analysis followed by Bonferroni’s multiple comparison test. The percentage of CLL in the blood at one time point (Figure 2G, Figure 3G and Figure 4I) was tested with the unpaired t test. To test significance in the adoptive transfer models (Figure 2J, Figure 3J and Figure 4L), we performed two-way ANOVA followed by Bonferroni’s multiple comparison test. A *p*-value lower than 0.05 was considered statistically significant.

## 3. Results

### 3.1. RNA Sequencing of CD5+CD19+ Leukemic B Cells from Eµ-TCL1 Mice

The development of leukemia involves the regulation of complex transcriptional programs allowing the cancer cells to proliferate, benefit from the support of the microenvironment, and escape the anti-tumor immune response [31,32]. In order to understand the biology of the leukemic cells in vivo, we performed RNA sequencing analysis of B cells sorted from control C57BL/6 (WT) and leukemic Eµ-TCL1 (TCL1) mice. Principal component analysis and correlation analysis showed a distinct transcriptional profile in leukemic B cells when compared with WT B cells, the gene expression profiles of leukemic B cells being more variable (Figure 1A and Supplementary Materials Figure S1A). Cluster analysis identified six clusters of deregulated genes in TCL1 B cells (K-means clustering, Figure 1B and Supplementary Materials Figure S1B–C). In particular, a decreased expression of genes involved in immune functions and lymphocyte activation was noticed, while genes involved in cell cycle, cell adhesion, locomotion, and cytoskeleton were upregulated. About 2457 genes were found statistically differentially expressed between WT and TCL1, with 1416 genes being more expressed in leukemic B cells, and 1041 genes being more expressed in WT B cells (Figure 1C,D).

Next, we performed a Gene Set Enrichment Analysis (GSEA) to identify hallmark pathways regulated in TCL1 and WT B cells. Among the 33 gene sets upregulated in TCL1 B cells and presenting a nominal enrichment score (NES > 1), we selected 22 gene sets as relevant for immune cells (Table 1). The most regulated gene sets were related to the TFs MYC, E2F, and TP53 and to several metabolic pathways linked to energy production, cholesterol/lipids, and glucose metabolism, indicating highly activated and proliferating cells. In addition, many signaling pathways involved in immune functions and cytokine secretion were enriched (IL-2/STAT5, mTORC1, IL-6/JAK/STAT3, and β-catenin). Finally, we observed the regulation of the two microenvironment-modulated pathways (hypoxia and xenobiotic stress) regulated by the TFs HIF family members and AHR, respectively (Figure 1E and Table 1). These two pathways are often regulated in cancer by a reduced oxygen concentration (hypoxia) and by tryptophan derivatives such as kynurenine [20]. Protein–protein interaction analysis (STRING) confirmed the tight connection between the transcription factors regulating these pathways (Figure 1F). 

While the response to hypoxia and glycolysis are both mediated by HIF-1α [33], the response to xenobiotic stress is complex and more diverse, in terms of transcriptional regulation. To confirm the potential role of AHR in TCL1 leukemic cells, we established a murine B cell-specific AHR gene signature from the publicly available gene expression dataset GSE80953 [34]. A list of 153 genes was identified as induced by the AHR agonist TCDD in murine B cells and used as a gene set database to run a GSEA. The analysis confirmed the enrichment of the AHR gene signature in TCL1 leukemic cells compared to normal B cells (NES = 1.16, FDR = 0.1, Figure 1G).

Then, we performed a transcription factor enrichment analysis (TFEA) with the online tool ChEA3 to better understand the regulation of transcriptional programs in leukemic cells. Again, both AHR and HIF-1α were listed among the top 50 TFs contributing to the regulation of DEGs in leukemic B cells (ranked 37 and 43 over 1632 TFs, respectively, Table 2). A second analysis with the ISMARA tool detected the use of specific and different TFs in WT and TCL1 B cells. For example, TAF1 was specifically used in WT cells, while Fos was more used by leukemic TCL1 cells (Supplementary Materials Figure S1D). Concerning HIF-1α and AHR, an enrichment of binding motifs was detected in DEGs from TCL1 leukemic cells compared to WT cells (Figure 1H).

Given the enrichment of HIF-1α and AHR gene signatures in Eµ-TCL1 mice compared to WT mice, their important role in cancer, and their ability to heterodimerize with the same binding partner called HIF-1β/Arnt [35,36], we decided to study the role of both AHR and HIF-1α in leukemogenesis in the Eµ-TCL1 murine model of CLL.

### 3.2. HIF-1α Knock-Out in Murine CLL Cells Does Not Impact Leukemogenesis 

HIF-1α is important in many cancers, also in CLL. However, the impact of HIF-1α on CLL leukemogenesis in vivo has not been fully investigated yet. For this purpose, we generated conditional transgenic knock-out mice using the Cre-*loxP* system (Figure 2A). First, by crossing *CD19^Cre/WT^* mice with *Hif1a^fl/fl^* mice, we ensured the gene knock-out specifically in B cells, then we crossed the newly generated strain (*CD19^Cre/WT^ Hif1a^fl/fl^*) with Eµ-TCL1, creating the Eµ-TCL1 *CD19^Cre/WT^ Hif1a^fl/fl^* transgenic model. In order to validate if HIF1α knock-out in Eµ-TCL1 *CD19^Cre/WT^ Hif1a^fl/fl^* was efficient compared to the control mice (Eµ-TCL1 *CD19^Cre/WT^ Hif1a^WT/WT^*), we isolated DNA, RNA, and proteins from CD19+ cells. As shown in Figure 2B–D and Supplementary Materials Figure S2, the knock-out of HIF-1α in the mice was validated on all levels. To further investigate the impact of HIF-1α knock-out in leukemic cells, we monitored CLL development in the transgenic mice over several months. In the Eµ-TCL1 *CD19^Cre/WT^Hif1α^fl/fl^* mouse model, we could not observe any difference in the survival compared to the control mice (Figure 2E). This finding was in accordance with the percentage of the circulating neoplastic cells in the peripheral blood, progressively accumulating over time (Figure 2F,G). Additionally, we performed RNA sequencing of leukemic B cells, comparing Eµ-TCL1 *CD19^Cre/WT^ Hif1a^fl/fl^* mice with the control mice. Here, we could not observe any major differences in the gene expression profiles (Figure 2H,I and Supplementary Materials Table S1). 

To investigate the importance of HIF-1α in healthy B cells in the CLL tumor microenvironment, we adoptively transferred Eµ-TCL1 CLL cells into mice lacking HIF-1α in healthy B cells, *CD19^Cre/WT^ Hif1a^fl/fl^*. Here, we could not observe any difference in the development of CLL in knock-out compared to control mice (Figure 2J).

These results led us to conclude that in this model of CLL, HIF-1α seems not to be an important transcription factor in healthy and leukemic B cells for proliferation in WT mice and leukemogenesis in the transgenic murine model.

### 3.3. AHR Knock-Out in Murine CLL Cells Does Not Impact Leukemogenesis

The transcription factor AHR plays a role in B cell proliferation and development [23,37]. Here, we investigated the importance of AHR in the development of leukemic B cells. To do so, we used the same strategy as described above for the HIF-1α knock-out model (Figure 3A). In accordance with this, DNA, RNA, and proteins from CD19+ cells of the Eµ-TCL1 *CD19^Cre/WT^Ahr^fl/fl^* mice were isolated for the validation of the knock-out. Compared to the control mice Eµ-TCL1 *CD19^Cre/WT^Ahr^WT/WT^*, the knock-out mice showed the excised allele of exon 2 of Ahr at the DNA level in B cells, no expression of exon 2 at the RNA level, and absence of the corresponding protein (Figure 3B–D and Supplementary Materials Figure S2), confirming that the knock-out was efficient.

Next, in order to analyze the impact of AHR knock-out in leukemic B cells, CLL development in the new transgenic model (Eµ-TCL1 *CD19^Cre/WT^ Ahr^fl/fl^*) and corresponding control (Eµ-TCL1 *CD19^Cre/WT^ Ahr^WT/WT^*) was monitored over several months. Eµ-TCL1 *CD19^Cre/WT^ Ahr^fl/fl^* mice compared to the control mice did not show any difference in survival (Figure 3E). In addition, the percentage of the AHR knock-out CLL cells in the blood did not differ compared to the control at different time points (Figure 3F,G). RNA sequencing performed on CLL cells did not reveal any relevant changes in the RNA profile of the knock-out mice compared to the control (Figure 3H, I and Supplementary Materials Table S2).

Furthermore, in order to investigate whether AHR knock-out in normal B cells could impact CLL development, we injected Eµ-TCL1 CLL cells into mice lacking AHR in normal B cells (*CD19^Cre/WT^ Ahr^fl/fl^*). As observed for the transgenic model, we could not detect any difference in CLL development for the adoptive transfer in either *CD19^Cre/WT^ Ahr^fl/fl^* or *CD19^WT/WT^ Ahr^fl/fl^* recipient mice (Figure 3J).

Based on the results of our AHR knock-out experiments in leukemic and in healthy B cells, AHR does not appear to be an essential factor in CLL development. 

### 3.4. AHR and HIF-1α Double Knock-Out Does Not Interfere with Murine CLL Development

The dimerization partner ARNT/HIF1β connects the pathways of HIF-1α and AHR, as both TFs share it in order to bind to DNA. As we could not observe a difference by removing these two TFs separately, we decided to investigate the potential synergistic role of HIF-1α and AHR in leukemogenesis by knocking them out together in our CLL murine model.

To achieve this, we crossed the *Ahr^fl/fl^* and *Hif1α^fl/fl^* mice, and the resulting strain was further crossed with the Eµ-TCL1 *CD19^Cre/WT^ Hif1α^fl/fl^* model (Figure 4A). This strain was further bred with *Hif1α^fl/fl^ Ahr^fl/fl^* mice. After obtaining the double knock-out mice (Eµ-TCL1 *CD19^Cre/WT^ Hif1a^fl/fl^ Ahr^fl/fl^*), we validated the conditional knock-out at DNA (Figure 4B,C), RNA (Figure 4D,E), and protein (Figure 4F and Supplementary Materials Figure S2) levels in CD19+ B cells. Similar to the previous results in single conditional knock-out mice, we could not observe any difference between Eµ-TCL1 *CD19^Cre/WT^ Hif1a^fl/fl^ Ahr^fl/fl^* and the control mice (Figure 4G). Although a slight increase in CLL cells in the blood of Eµ-TCL1 *CD19^Cre/WT^ Hif1α^fl/fl^ Ahr^fl/fl^* mice was visible at month 6, this difference evened out in the following months (Figure 4H,I). RNA sequencing of CLL cells did not reveal differences in gene expression between Eµ-TCL1 *CD19^Cre/WT^Hif1α^fl/fl^ Ahr^fl/fl^* and the control mice (Figure 4J,K and Supplementary Materials Table S3). Finally, adoptive transfer of Eµ-TCL1 CLL cells into mice carrying the double knock-out in normal B cells showed no differences in disease development (Figure 4L).

Taken together, the deletion of the transcription factors HIF1α and AHR does not appear to be crucial in our murine CLL models. It is possible that the two TFs are not involved in CLL development and are not crucial for the function of healthy B cells in the clearance of CLL.

## 4. Discussion

In recent years, targeted therapy has shown to be highly efficient against CLL, improving patients living conditions and overall survival. Most of the current treatments are focusing on anti-apoptotic molecules (Bcl-2) and BCR-signaling kinases (BTK, PI3K), due to the highly stimulated state of CLL cells in the microenvironment. Innovative molecular therapy also aims to target leukemic cells’ vulnerabilities more precisely. The use of conventional DNA-damage-based chemotherapy drugs in the past decades and the understanding of molecular mechanisms led to the discovery of potential new targets (e.g., WEE1 kinase) [38].

As heavily mutated and regulated in cancers, transcription factors naturally attracted attention for the development of new therapeutics. Around 300 TFs were described as oncogenes and could therefore become druggable candidates (e.g., TP53, MYC, and Stat family members) [39]. AHR and HIF-1α are not oncogenes but are crucial regulators of transcription programs in the microenvironment as sensors/responders to microenvironment conditions in both cancer and immune cells [40].

Previous research has shown that AHR and HIF-1α are important in hematological malignancies [13,41]. The impairment of HIF-1α and AHR provided promising results against leukemia and myeloma. Indeed, HIF-1α silencing by shRNA impaired the homing of CLL cells to the bone marrow and spleen, and the chemical inhibition of HIF-1α with EZN-2208 prolonged the survival of mice challenged with MEC-1 cells [26]. In addition, the FDA-approved AHR antagonist clofazimine showed high efficacy in a transgenic model of multiple myeloma [42]. However, these observations are not sufficient to understand the exact mechanism of action and the cells targeted by the therapy. Whether the molecule directly kills cancer cells or impacts the immune microenvironment should be understood to refine treatments and propose new combinatory therapeutics for improved patient outcome. Therefore, we made use of transgenic murine models to specifically study the importance of both TFs in in leukemic cells in the context of CLL. We used the well-established Eµ-TCL1 murine model, where we showed that both AHR and HIF-1α transcriptional programs are enriched. We crossed this model with *Hif1a* or *Ahr* conditional knock-out mice to delete these genes only in CD19^+^ B cells and investigated their role in the leukemogenesis of CLL. The unexpected results in this article show that knocking-out these two transcription factors does not affect the development of CLL in vivo. The gene expression profile of leukemic cells compared to that of cells from the control group was also unaffected by both knock-outs. Although we cannot rule out that there might be a different expression profile at early stages of the disease, this does not affect the final outcome of CLL progression. In addition, using our adoptive transfer model of TCL1 CLL cells into *Hif1a* or *Ahr* conditional knock-out, we could infer their role in normal B cells in the leukemic microenvironment. Indeed, the role of B cells in the context of cancer is still poorly defined and needs more investigations to better elucidate B cells pro-tumoral or anti-tumoral impact in different types of cancer [43]. Here, we could show that knocking out AHR and HIF-1α in healthy B cells did not impact the progression of CLL, which led to the conclusion that AHR and HIF-1α do not play a crucial role either in the development of CLL B cells or in the function of B cells in the TME.

From the literature, HIF-1α seems to be an interesting new target for CLL therapy, as it regulates the interaction of CLL cells with the TME and is upregulated in unmutated immunoglobulin heavy-chain variable region genes (IGHV) and TP53-disrupted CLL patients [26,27]. Interestingly, Meng et al. showed that the HIF-1α pathway directly affects IL-10 production in B cells [33], a feature also used by CLL B cells to favor an immunosuppressive tumor microenvironment and increase their survival [44]. Despite this, deletion of HIF-1α in leukemic cells of the Eµ-TCL1 mouse model had no impact on disease development, leading to the hypotheses that the importance of HIF-1α could depend on the oncogenic drivers and/or on compensatory mechanism (e.g., HIF-2α and/or on other cells of the TME). Griggio et al. nicely showed that the combination of an HIF-1α inhibitor with ibrutinib, a BTK inhibitor, showed a synergistic cytotoxic effect in TP53-disrupted CLL cells [27]. Considering this, it would be interesting to further investigate the effect of TP53 mutation in the TCL1 mouse model including the HIF-1α knock-out. Further, the use of a HIF-1α inhibitor in a co-culture of CLL cells and stromal cells in vitro showed that the inhibition also affected the stromal cells, resulting in decreased transcriptional regulation of target genes in the stromal compartment [45]. Considering the intricate regulation of the immune response by HIF-1α/A2A adenosine receptor signaling pathways [46], it would be worth investigating HIF-1α and its inhibition in the tumor microenvironment, including T cells, regulatory T cells, and nurse-like cells.

Regarding AHR, it also plays an important role in carcinogenesis and other diseases, especially the IDO–Kyn–AHR axis [47]. IDO activity is increased in CLL patients [48]. Furthermore, AHR is important in B cell development by controlling cell proliferation and apoptosis [21,23] and is involved in regulatory B cell (Bregs) differentiation and in the regulation of IL-10 production [24]. As CLL cells and Bregs share similar functions [25], AHR appears as an interesting therapeutic target in CLL. Nevertheless, and similarly to HIF-1α, its deletion did not influence disease outcome in the Eµ-TCL1 mouse model. According to previous studies, AHR is an important player in the establishment of an immunosuppressive TME. Jitschin et al. showed that untreated CLL patients have increased IDO^hi^CD62L^hi^PD-L1^hi^HLA-G^hi^ CD11b^+^CD33^+^CD14^+^HLA-DR^lo^ monocytic cells, supporting an immune suppression [49]. In addition, Sadik et al. recently demonstrated that IL4I1, which catalyzes the formation of the AHR-activating ligand kynurenine, enhances CLL development due to a highly immunosuppressive TME [50]. Thus, increased AHR activation could influence disease outcome by an enhanced suppressive environment, by regulating the function of regulatory T cells (Tregs), and by turning the phenotype of effector CD8^+^ T cells into an exhausted one [10,50]. Hence, targeting AHR in the TME of the proliferative CLL niche might still represent an interesting therapeutic option. 

As HIF-1α and AHR share the dimerization partner ARNT/HIF1β, we could speculate on a reciprocal compensation, as for instance, both were shown to regulate IL-10 expression in B cells [24,33]. Therefore, we asked whether knocking out both factors would then be effective in decreasing CLL progression. However, as for single knock-outs, the survival of the mice did not differ compared to the control group. The percentage of CLL cells in the blood showed a slight increase in the double knock-out mice. However, this difference evened out when the disease was progressing. A possible explanation for CLL growth in the double knock-out might be the presence of an immunosuppressive tumor microenvironment. Indeed, CLL cells may overcompensate the loss of both HIF-1α and AHR, by another TF. For instance, Sp1 can regulate IL-10 production in CLL following BCR activation and signaling. It could supply CLL cells with enough survival signals and provide an IL-10-mediated suppression of the host immune system [44]. 

## 5. Conclusions

In conclusion, our results demonstrate that targeting HIF-1α or AHR by deleting them in CLL cells does not influence disease development in the Eµ-TCL1 mouse model. However, our results are not contradictory to previous results, as targeting the surrounding cells by specific inhibitors of these TFs could decrease the pro-survival signals for CLL cells and restore a more active anti-tumor immunity leading to reduced tumor burden. In vivo studies in relevant mouse models are therefore crucial to determine the importance of specific targets and also to investigate the effect of targeted inhibitors on tumor cells as well as on surrounding TME cells. This is crucial to validate interesting new targets and determine the mode of action of targeted treatments. 

## Figures and Tables

**Table 1:** Relevant Gene Sets enriched in leukemic B cells compared to normal B cells as revealed by RNA sequencing analysis and GSEA. NES, normalized enrichment score. NOM *p*-val, nominal *p*-value.

Rank	GeneSets	SIZE	NES	NOM *p*-Val
1	HALLMARK_MYC_TARGETS_V1	200	1.46	0.000
2	HALLMARK_CHOLESTEROL_HOMEOSTASIS	73	1.45	0.000
3	HALLMARK_GLYCOLYSIS	200	1.4	0.000
4	HALLMARK_E2F_TARGETS	200	1.4	0.185
6	HALLMARK_MYC_TARGETS_V2	57	1.38	0.000
7	HALLMARK_OXIDATIVE_PHOSPHORYLATION	197	1.35	0.000
8	HALLMARK_REACTIVE_OXYGEN_SPECIES_PATHWAY	49	1.34	0.000
9	HALLMARK_FATTY_ACID_METABOLISM	157	1.34	0.000
10	HALLMARK_G2M_CHECKPOINT	196	1.29	0.185
11	HALLMARK_UV_RESPONSE_UP	156	1.28	0.000
15	**HALLMARK_HYPOXIA**	198	1.22	0.000
16	HALLMARK_P53_PATHWAY	200	1.22	0.084
18	HALLMARK_IL2_STAT5_SIGNALING	199	1.2	0.094
20	HALLMARK_MTORC1_SIGNALING	199	1.18	0.287
21	HALLMARK_UNFOLDED_PROTEIN_RESPONSE	112	1.15	0.185
24	HALLMARK_MITOTIC_SPINDLE	199	1.13	0.269
25	HALLMARK_DNA_REPAIR	148	1.13	0.084
28	**HALLMARK_XENOBIOTIC_METABOLISM**	196	1.09	0.299
30	HALLMARK_APOPTOSIS	161	1.01	0.376
31	HALLMARK_IL6_JAK_STAT3_SIGNALING	87	1.01	0.381
32	HALLMARK_PROTEIN_SECRETION	96	1.01	0.472
33	HALLMARK_WNT_BETA_CATENIN_SIGNALING	41	1	0.362

**Table 2:** Top50 ranking of transcription factors whose putative transcriptional targets are most closely similar to those in the query set. CHEA3 results for transcription factors enrichment analysis (TFEA). Lower scores indicate more relevancy to the related transcription factor. Full query = 1632 TFs.

Rank	TF	Score	Rank	TF	Score
1	FOXM1	4.333	26	CEBPB	88.83
2	CENPA	5.0	27	NFE2L2	89.4
3	ZNF367	8.333	28	ARNTL2	94.67
4	PA2G4	15.5	29	JDP2	95.67
5	HMGA2	24.67	30	ZNF888	97.0
6	AHRR	26.5	31	BHLHE40	97.75
7	ZNF695	29.0	32	ZNF670	98.5
8	E2F7	29.2	33	OSR1	99.5
9	E2F1	33.83	34	GLMP	101.5
10	FOSL1	35.4	35	HMGN3	105.0
11	ELK3	49.0	36	ZNF93	106.7
12	FOXD1	51.5	37	**AHR**	107.4
13	MYBL2	55.17	38	PPARG	108.0
14	TFDP1	56.0	39	TAL1	109.0
15	E2F2	65.33	40	ATF3	110.3
16	RFX8	68.0	41	TEAD4	119.8
17	CENPX	68.0	42	NR1H3	120.0
18	KLF6	72.0	43	**HIF1A**	120.2
19	CREB3L2	75.33	44	VDR	120.8
20	ETV4	76.5	45	KLF9	123.8
21	ZNF878	78.0	46	GLIS2	126.3
22	E2F8	80.0	47	RELB	128.3
23	DNMT1	81.0	48	PLSCR1	131.0
24	NR2F6	85.5	49	TEAD3	132.7
25	KLF4	87.0	50	HLX	136.3

## Data Availability

The data presented in this study are openly available in the public functional genomics data repository GEO under the references GSE175564 and GSE179196.

## Supplementary Materials

The following are available online at https://www.mdpi.com/article/10.3390/cancers13184518/s1, Figure S1: RNA sequencing data of B cells from C57BL/6 and Eµ-TCL1 mice. Figure S2: Full gel pictures corresponding to Figures 2D, 3D and 4F, Table S1: Differentially expressed genes in leukemic cells from TCL1 *CD19^Cre/WT^Hif1a^fl/fl^* versus Eµ-TCL1 *CD19^Cre/WT^Hif1a^WT/WT^*, Table S2: Differentially expressed genes in leukemic cells from TCL1 *CD19^Cre/WT^Ahr^fl/fl^* versus Eµ-TCL1 *CD19^Cre/WT^Ahr^WT/WT^*, Table S3: Differentially expressed genes in leukemic cells from TCL1 *CD19^Cre/WT^Ahr^fl/fl^Hif1a^fl/fl^* versus Eµ-TCL1 *CD19^Cre/WT^Ahr^WT/WT^Hif1a^WT/WT^*.
