# Supplementary material for: The Tumor Microenvironment-Dependent Transcription Factors AHR and HIF-1α Are Dispensable for Leukemogenesis in the Eµ-TCL1 Mouse Model of Chronic Lymphocytic Leukemia
Source: Cancers (Basel). 2021 Sep 8;13(18):4518. doi: 10.3390/cancers13184518 (PMC8466120; doi:10.3390/cancers13184518)
Supplement: Supplementary file 1 [file cancers-13-04518-s001.zip › cancers-1368431-SI.pdf]

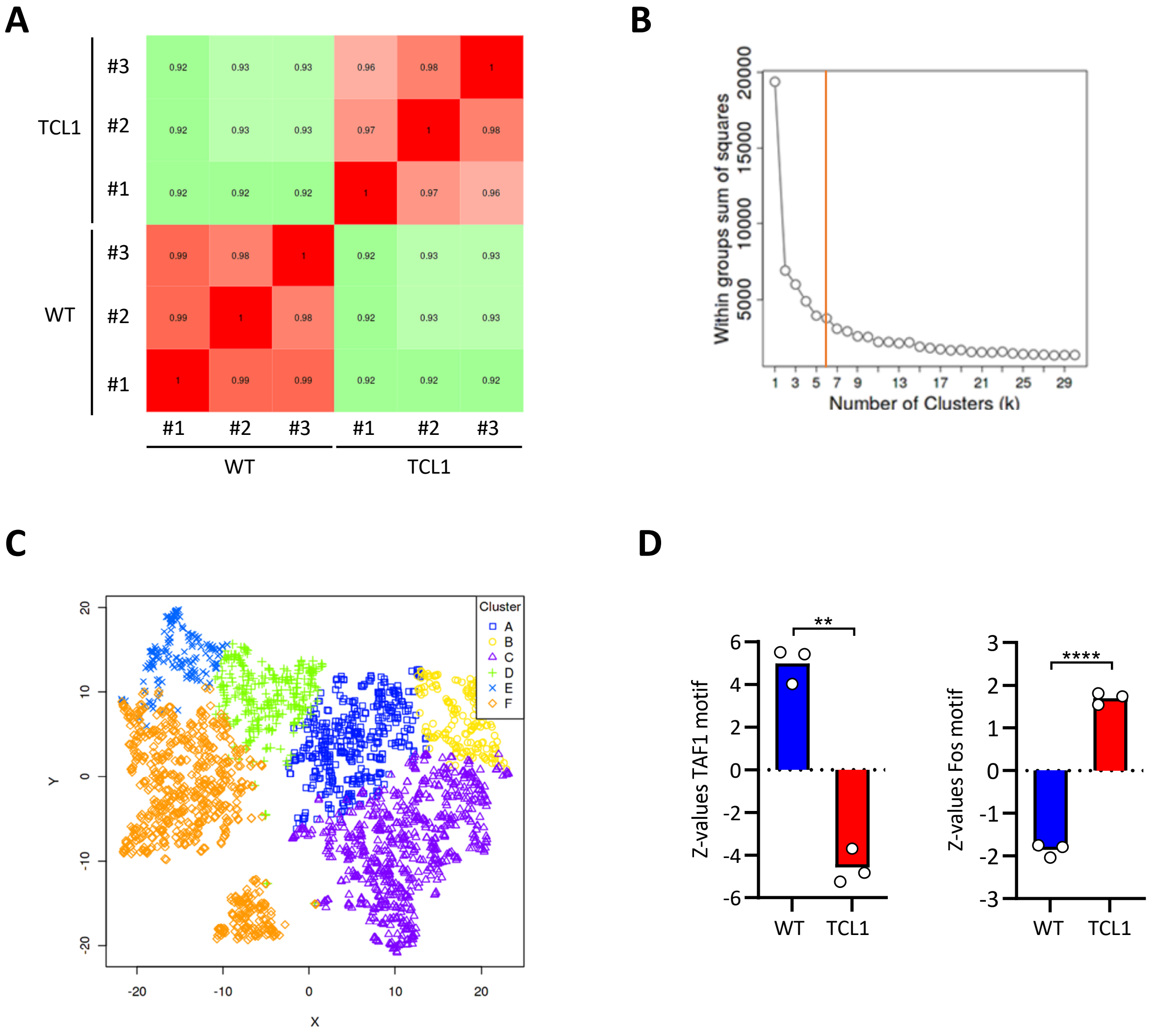

<u>Supplemental Figure 1</u>: RNA sequencing data of B cells from C57BL/6 and Eµ-TCL1 mice. **(A)** Correlation matrix for all biological replicates. **(B)** Estimate of the number of clusters (K value) using the elbow method. **(C)** Mapping of the top genes to k-means clusters with the dimension reduction algorithm t-SNE. **(D)** Transcription factor activity (Z-values, ISMARA) for TAF1 and Fos motifs among WT and TCL1 B cells.

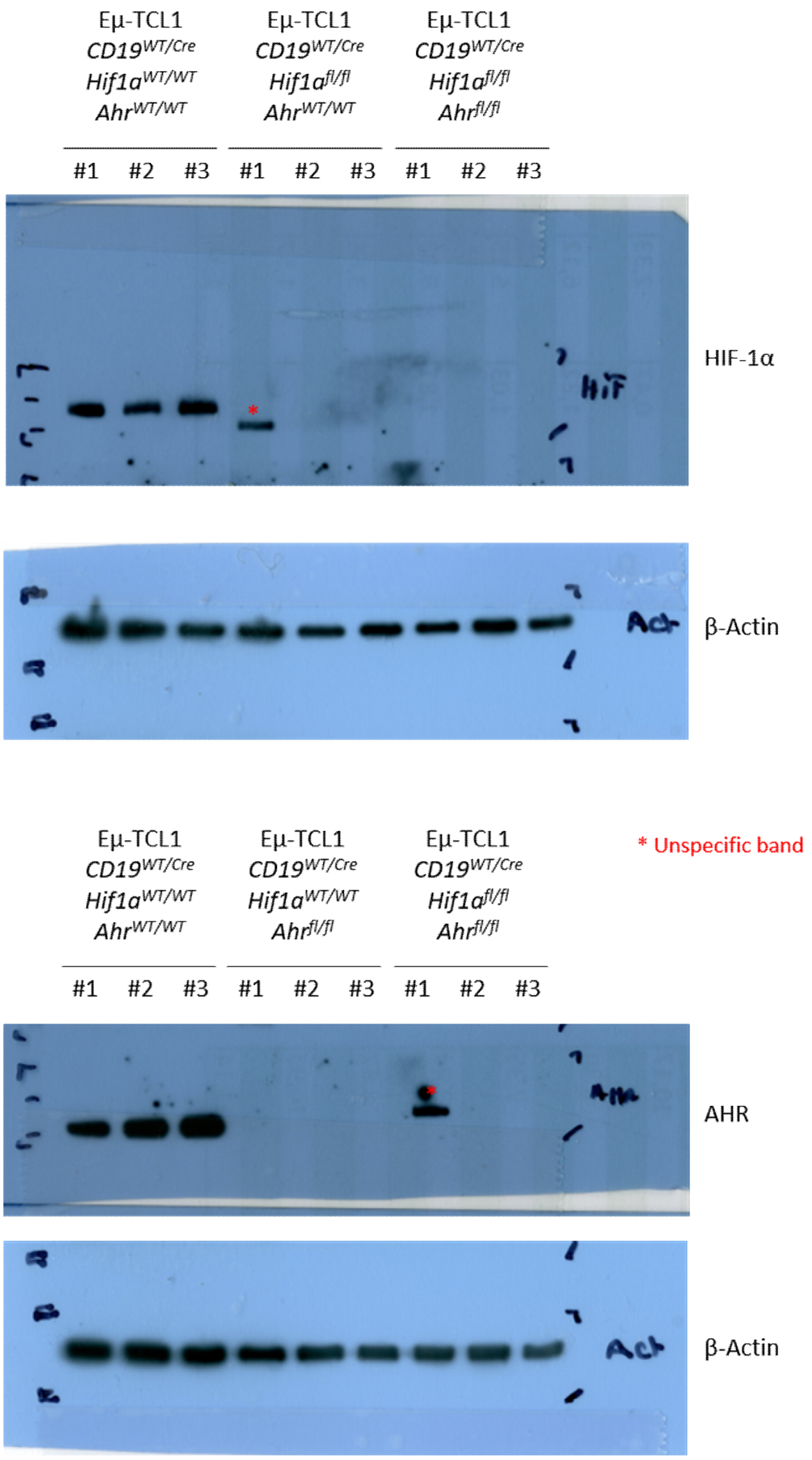

Supplemental Figure 2: Full gel pictures corresponding to Figure 2D, 3D, and 4F

Gene	logFC	FDR
Gstt1	6.47	5.487e-09
Prdm16	9.27	0.0001
Mcts2	3.69	0.0025
Igkv12-89	5.61	0.0043
Ighv1-55	7.14	0.0052
Cntln	6.60	0.0081
Scfd1	1.33	0.0248
Mnat1	1.35	0.0248
Peak1os	-4.38	0.0350
Gm20767	-1.57	0.0350
St6gal1	-1.97	0.0350
Cd36	-2.40	0.0350
Calcrl	4.45	0.0387
Ctnna1	3.21	0.0471

Supplemental Table 1. Differentially expressed genes in leukemic cells from TCL1 $\textit{CD19}^{Cre/WT}\textit{Hif1a}^{fl/fl}$ versus Eμ-TCL1 $\textit{CD19}^{Cre/WT}\textit{Hif1a}^{WT/WT}$.

Gene	logFC	FDR
Leprotl1	2.33	0.0185
Alox5ap	-2.65	0.0185
Prdm16	5.22	0.0265
Icos	-4.30	0.0342

Supplemental Table 2. Differentially expressed genes in leukemic cells from TCL1 $\textit{CD19}^{Cre/WT}\textit{Ahr}^{fl/fl}$ versus Eμ-TCL1 $\textit{CD19}^{Cre/WT}\textit{Ahr}^{WT/WT}$.

Gene	logFC	FDR
none		

Supplemental Table 3. Differentially expressed genes in leukemic cells from TCL1 $\textit{CD19}^{Cre/WT}\textit{Ahr}^{fl/fl}\textit{Hif1a}^{fl/fl}$ versus Eμ-TCL1 $\textit{CD19}^{Cre/WT}\textit{Ahr}^{WT/WT}\textit{Hif1a}^{WT/WT}$.
